# The Transcription Factor EGR1 Localizes to the Nucleolus and Is Linked to Suppression of Ribosomal Precursor Synthesis

**DOI:** 10.1371/journal.pone.0096037

**Published:** 2014-05-01

**Authors:** Donatella Ponti, Gian Carlo Bellenchi, Rosa Puca, Daniela Bastianelli, Marella Maroder, Giuseppe Ragona, Pascal Roussel, Marc Thiry, Dan Mercola, Antonella Calogero

**Affiliations:** 1 Department of Medico-Surgical Sciences and Biotechnologies, University of Rome La Sapienza, Latina, Italy; 2 Institute of Genetics and Biophysics ABT, Via Pietro Castellino, Napoli, Italy; 3 RNA Biology, FRE 3402 CNRS-Universitè Pierre et Marie Curie, Paris, France; 4 Unit of Cell Biology, GIGA-Neuroscience, University of Liege, CHU SartTilman, Liege, Belgium; 5 Department of Pathology and Laboratory Medicine, University of California, Irvine, California, United States of America; University of Dundee, United Kingdom

## Abstract

EGR1 is an immediate early gene with a wide range of activities as transcription factor, spanning from regulation of cell growth to differentiation. Numerous studies show that EGR1 either promotes the proliferation of stimulated cells or suppresses the tumorigenic growth of transformed cells. Upon interaction with ARF, EGR1 is sumoylated and acquires the ability to bind to specific targets such as PTEN and in turn to regulate cell growth. ARF is mainly localized to the periphery of nucleolus where is able to negatively regulate ribosome biogenesis. Since EGR1 colocalizes with ARF under IGF-1 stimulation we asked the question of whether EGR1 also relocate to the nucleolus to interact with ARF. Here we show that EGR1 colocalizes with nucleolar markers such as fibrillarin and B23 in the presence of ARF. Western analysis of nucleolar extracts from HeLa cells was used to confirm the presence of EGR1 in the nucleolus mainly as the 100 kDa sumoylated form. We also show that the level of the ribosomal RNA precursor 47S is inversely correlated to the level of EGR1 transcripts. The EGR1 iseffective to regulate the synthesis of the 47S rRNA precursor. Then we demonstrated that EGR1 binds to the Upstream Binding Factor (UBF) leading us to hypothesize that the regulating activity of EGR1 is mediated by its interaction within the transcriptional complex of RNA polymerase I. These results confirm the presence of EGR1 in the nucleolus and point to a role for EGR1 in the control of nucleolar metabolism.

## Introduction

The early growth response gene EGR1 codes for a zinc finger nuclear factor involved in the transcriptional regulation of responses to a wide number of proliferative, differentiation and stress stimuli [1]–[3]. In particular, EGR1 regulates key genes controlling the growth and division of cancer cells, including p53 and PTEN, which form a regulatory network [4]–[6]. Working coordinately, they can suppress transformed cell growth [7]. In addition, EGR1 can suppress uncontrolled cell proliferation by p53 independent mechanism(s) [7]. As a consequence, EGR1 is often downregulated or lost in human cancer tissues and cell lines [8]–[10]. Reexpression of EGR1 in EGR1-deficient transformed cell lines limits cancer cell growth and tumorigenicity, suggesting a role for EGR1 in promoting the growth arrest of transformed cell variants [11]–[12], [3] and it also augments the sensitivity to chemotherapeutic treatments [13]–[15].

Recent studies have suggested a role for altered proto-oncogenes and tumor suppressor genes in the subversion of control mechanisms regulating ribosome biogenesis [16]. In eukaryotic cells the assembly of rRNA with the ribosomal proteins and the many small nucleolar RNAs (snoRNAs) takes place in the nucleolus. It is a highly coordinated process involving both transcriptional and post-transcriptional events that together control ribosomal protein synthesis. Ribosomal protein synthesis is increased in tumor cells [17], and it is crucial for tumor progression [18]. In fact, particularly aggressive phenotypes of cancer are associated with changes in nucleolar morphology including increased size, and require elevated rates of biosynthesis and higher levels of rRNA transcription [19]–[20]. Cell cycle checkpoints are important in coordinating ribosome production with cell-cycle progression [21]–, as also several tumor suppressor agents such as P53, PTEN, RB and ARF. The ARF protein has been shown to regulate the cell cycle through both p53-dependent and p53-independent pathways. In addition to the ARF-MDM2-p53 pathway, several partners of ARF have recently been described that could partecipate in alternative regulatory pathways such as MYC. In particular, the ARF-MYC interaction is crucial for driving the MYC-induced synthesis of EGR1, which in turn is essential for mediating the induction of p53-independent apoptosis [23]. Moreover, it is known that ARF is a negative regulator of rRNA transcription and maturation. For instance, ARF binds to and inhibits the phosphorylation of the upstream binding transcription factor UBF1 [24]. ARF also promotes the sumoylation of several ARF interacting proteins such as the topoisomerase I, MDM2, p53 and EGR1 itself [25]–[28]. The ARF-mediated sumoylation of EGR1 is strictly required for PTEN activation in vivo, which in turn is directly involved in the regulation of cell size and protein synthesis [29]–[30]. Sumoylation is a post-translational modification that may alter the cellular trafficking, thus affecting the subcellular localization of the modified proteins.

Based on the above relationships, we asked whether the transcription factor EGR1 also could play a role in nucleolar metabolism. Here we provide the molecular evidence that EGR1 localizes to the nucleolus. We also found that the level of ribosomal RNA precursors varies inversely with level of EGR1 transcripts. In fact, by treating the cells with siRNA specific for EGR1 we observed a significant increase in the production of 47S pre-rRNA in the HeLa cell line. Conversely, by increasing the level of EGR1 transcription we observed a significant reduction of the synthesis of 47S pre-rRNA. The effect of EGR1 on RNA polymerase I activity is linked to ARF because it cannot be reproduced in *ARF −/−* NIH 3T3 cells but can be restored after ARF re-expression. Like ARF, also EGR1 binds directly to UBF, which is required to recruit the transcription complex at the rRNA promoter. Taken together, these results suggest that the anti-proliferative properties of EGR1 might also stem from its association with protein(s) involved in the control of RNA polymerase I activity within the nucleolus.

## Materials and Methods

### Cell Cultures

The cell lines (HeLa, NH 3T3, 293T and A172) were grown in DMEM supplemented with 1% nonessential amino acids, 1% L-glutamine, 100 IU/ml penicillin, 100 IU/ml streptomycin and FBS (Sigma-Aldrich St. Louis, Mo, USA) 10% or 0.2% (serum starvation) at 37°C in 5% CO2 humidified atmosphere in air. HeLa cells (ATCC CCL2), NIH 3T3 (ATCC CR-1658), 293 T (ATCC CRL-1573), A172 (ATCC CRL-1620) are provided by American Type Collection, Rockville, MD.

### Confocal Analysis

The cells were cultured in DMEM in 10% FBS or 0.2% FBS for 18h on Nuc Lab-Tek II chamber slide (Sigma-Aldrich). Cells were washed with PBS, fixed for 15 min with 4% paraformaldehyde (Sigma-Aldrich St. Louis, Mo, USA), washed with PBS, permeabilized with 0.5% Triton X-100 for 10 min and blocked for 40 min with 0.2% gelatin. All incubations with primary antibodies were performed in PBS-Triton X-100 overnight at 4°C. The following primary antibodies were used for immunofluorescence: rabbit polyclonal against N-terminal of EGR1 (4153, Cell Signaling Technology, Danvers, MA, USA), mouse monoclonal antibody anti-fibrillarin (ab4566, Abcam, Cambridge, MA, USA), mouse monoclonal antibody anti-B23 (ab10530, Abcam) and mouse monoclonal anti-UBF antibody (sc-13125, Santa Cruz Biotechnology, Dallas, TX, USA). Primary antibodies were diluted at 1∶200. Cells were than washed 3 × 5 min and incubated with secondary antibodies, Alexa Fluor mouse 594 and Alexa Fluor rabbit 488 diluted 1∶1000. Confocal analysis was performed with a Leica SP2. Transcription of rDNA genes was inhibited by supplementing the medium with 0.04 µg/ml actinomycin D (Sigma-Aldrich St. Louis, Mo, USA) for 1h at 37°C the cells were than fixed and stained. The analysis was performed by immunofluorescence microscopy (LEICA DM4000B).

### Electron Microscopy

To study the intranuclear localization of the transcription factor EGR1, we realised several immunogold labelings on ultrathin sections of HeLa cells. Cells were fixed for 1 h at 4°C in 4% formaldehyde in 0.1 M Sörensen’s buffer (pH 7.4), dehydrated through graded ethanol solutions, and embedded in Lowicryl K4M as in [31]. Ultrathin sections of Lowicryl K4M-embedded cells were incubated for 30 min in PBS (0.14 M NaCl, 6 mM Na_2_HPO_4_, 4 mM KH_2_PO_4_, pH 7.2) containing normal goat serum (NGS) diluted 1/30 and 1% BSA, then rinsed with PBS containing 1% BSA. After a 4 h incubation with rabbit polyclonal antibody against N-terminal of EGR1 (Cell Signaling Technology, Danvers, MA, USA) diluted 1/2.5 in PBS containing 1/50 NGS and 0.2% BSA, the sections were washed with PBS containing 1% BSA, and incubated for 60 min with goat anti-rabbit IgG coupled to colloidal gold (10 nm in diameter) (Amersham Life Science) diluted 1/40 with PBS (pH 8.2) containing 0.2% BSA. After washing with PBS containing 1% BSA, the sections were rinsed in deionized water. Finally, the ultrathin sections were mounted on nickel grids, and stained with uranyl acetate and lead citrate before examination in a Jeol CX 100 II transmission electron microscope at 60 kV. A control experiment was carried out, in which the primary antibodies were omitted.

### Immunoblotting and Immunoprecipitation

Western blot analysis was performed using nuclear and nucleolar extracts. The following primary antibodies were used for immunoblotting: rabbit polyclonal anti-EGR1 (sc-101, Santa Cruz Biotechnology, Dallas, Tx, USA) mouse monoclonal antibody anti-fibrillarin (ab4566), monoclonal anti-Sumo1 (SAB4200189, Sigma-Aldrich St. Louis, Mo, USA) and mouse monoclonal anti-UBF antibody (sc-13125). The secondary antibodies used for western blot are anti-mouse and anti-rabbit (GE Healthcare Bio-Sciences, Piscataway, NJ, USA) (dilution 1∶10000). *Whole extracts.* Total extract were prepared from subconfluent cultures by resuspending cells in RIPA-Buffer (20 mM Hepes, pH 6.8, 5 mM KCl, 5 mM MgCl_2_, 0.5% NP-40, 0.1% sodium deoxycholate, protease inhibitor (Sigma), 0.1 mM phenylmethylsulfonyl fluoride) after incubation for 30 min at 0°C centrifuge at 10000 rpm × 15 min 4°C. *Nuclear extracts:* cells at 80% of confluence were washed twice with PBS, and incubated in NE1 buffer (10 mM Hepes pH 8.0, 1.5 mM MgCl_2_, 10 mM KCl, 1 mM DTT) for 15 min at 4°C. Homogenization of the cells was performed using a Dounce homogenizer and the lysate was centrifuged at 12.000 rpm for 5 min at 4°C. The nuclear pellet was resuspended in NE2 buffer (20 mM Hepes pH 8.0, 1.5 mM MgCl_2_, 25% glycerol, 420 mM NaCl, 0.2 mM EDTA, 1 mM DTT and 0.5 mM PMSF) and incubated for 30 min at 4°C (41). Finally, the supernatant was cleared from the insoluble nucleolar fraction by centrifuging for 2 min at 12.000 rpm and was diluted 1∶4 with water. *Nucleolar extracts:* the nucleolar extracts were prepared as followed. Actively growing cells were washed in cold PBS and lysed at 4°C in a hypotonic buffer (10 mM Tris-HCl, pH 7.4, 25 mM NaCl, and 3 mM MgCl_2_). Lysis performed with the Tissue Master 125 homogenizer (OMNI international) was stopped when the nuclei appeared free of cytoplasmic components as assessed by phase microscopy. The nuclei were then collected by centrifugation at 1200 g for 5 min, resuspended in 10 mM Tris-HCl, pH 7.4, 10 mM NaCl, 10 mM MgCl_2_, 0.25 M sucrose and purified on a 0.88 M sucrose cushion prepared in 10 mM Tris-HCl, pH 7.4, 10 mM NaCl, 1.5 mM MgCl_2_ at 1200 g for 10 min. The nucleoli were isolated by sonication of nuclei suspended in 10 mM Tris-HCl, pH 7.4, 10 mM NaCl, 1.5 mM MgCl_2_, 0.34 M sucrose, and 0.25% NP40. The nucleoli were then purified on a 0.88 M sucrose cushion prepared as previously at 2000 g for 20 min. The purified nucleoli were washed by suspension in 10 mM Tris-HCl, pH 7.4, 10 mM NaCl, 1.5 mM MgCl_2_, 0.34M sucrose and centrifugation at 2000 g for 5 min. All steps were performed at 4°C and all the solutions contained a cocktail of protease inhibitors (complete, Roche Molecular Diagnostics, Pleasanton, CA, USA). For immunoprecipitation experiments, an equal amount of whole protein extracts (150 µg) from transfected HeLa cells were immunoprecipitated with anti-UBF antibody (sc-13125, Santa Cruz Biotechnology, Dallas, TX, USA) or with anti-EGR1 antibody, or with secondary antibody (IgG anti-mouse) 3h at 4°C in binding buffer (50 mM Tris pH 7.8 and 150 mM NaCl) using magnetic beads (Merk Millipore, Darmstadt, Germany) as recommended by the manufacturer. Total extract were prepared from subconfluent cultures by resuspending cells in RIPA-Buffer (20 mM Hepes, pH 6.8, 5 mM KCl, 5 mM MgCl_2_, 0.5% NP-40, 0.1% sodium deoxycholate, protease inhibitor (Sigma-Aldrich St. Louis, Mo, USA), 0.1 mM PMSF (phenylmethylsulfonyl fluoride, Sigma-Aldrich St. Louis, Mo, USA) and 25 mM NEM (N-Ethylmaleimide, Sigma-Aldrich St. Louis, Mo, USA). After incubation for 30 min at 4°C the extract were centrifuged at 10000 rpm × 15 min at 4°C. The beads were washed three times with binding buffer and incubated at 95°C for 10 min with 50 µl of 2×SDS loading buffer. Precipitated proteins were resolved by 10% SDS-PAGE and immunoblotted with anti-EGR1 or anti-UBF antibodies. The membrane was incubated for the primary antibodies over night in 5% dry milk at 4°C. The incubation with secondary antibodies (1h at room temperature) followed by ECL reaction (Amersham, Buckinghamshire, UK) according to the manufacturer’s instructions. The membrane was exposed to Kodak film (Amersham Hyperfilm ECL). All experiments have been done in triplicate.

### qRT-PCR

RNA extraction was performed after EGR1 silencing and EGR1 overexpression. Total RNA extraction and cDNA preparation were accomplished using RNeasy (Qiagen, Valencia, CA) and Superscript III RT (Invitrogen, Grand Island, NY USA) according to the manufacturer’s recommendations. For silencing, EGR1 pre-designed siRNA (Invitrogen, Grand Island, NY USA, 4390822 for Hela cells and 4390817 for NIH 3T3 or scrambled sequence RNA oligonucleotide, Negative Control siRNA 4390846) were transiently transfected at 10 nM or 15 nM into HeLa and NIH 3T3 cells using High-Perfect Transfecting Agent (Quiagen, Valencia, CA) following the Quiagen protocol. Full length EGR1 (1–543) and deleted forms were cloned into pEGFP (Clontech, Mountain View, CA, USA). Each construct was confirmed by DNA sequencing (3130 Genetic Analyzer, Applied Biosystem). The experiments of DNA transfection in NIH 3T3 were performed with 2 µg of DNA for each expression vector. p19ARF expression vector correspond to Image ID 5342027 clone. Proteins expression was verified by fluorescence.

Quantitative RT-PCR was performed using Fast SYBR Green Master mix and the StepOnePlus real-time PCR system (both from Applied Biosystems). Each experiment was performed in triplicate and is expressed as mean ± SEM. Experiments were independently repeated three times. Gene expression levels were quantified from real-time PCR data by the comparative threshold cycle (CT) method using 18S as an internal control gene. The following gene-specific primers were used: EGR1: FW 5′-AGCCCTACGAGCACCTGAC-3′ and Rev 5′-GGTTTGGCTGGGGTAACTG-3′ (for human gene); FW 5′-CCTATGAGCACCTGACCACA-3′ and Rev 5′-TCGTTTGGCTGGGATAACTC-3′ (for mouse gene). 47S: FW 5′-TGTCAGGCGTTCTCGTCTC-3′ and REV 5′-gagagcacgacgtcaccac-3′ (for human gene) and FW 5′-CCCGAGTGCATTTCTTTTTG -3′ and Rev 5′-TGGACACCACAGACAGGAGT -3′ (for mouse gene). P300: FW 5′-GGTCAAGCTCCAGTGTCTCAA-3′ and Rev 5′- CCCTGGAGGCATTATAGGAGA-3 (for human gene). 18S: FW 5′-GCAATTATTCCCCATGAACG-3′ and REV 5′GGGACTTAATCAACGCAAGC-3′ (for human gene) and FW 5′-AAATCAGTTATGGTTCCTTTGGTC-3′ and Rev 5′-GCTCTAGAATTACCACAGTTATCCAA -3′ (for mouse gene).

### Chromatin Immunoprecipitation (ChIP)

Hela cells were transfected with full length EGR1 expression vector or pEGFP empty vector using lipofectamine 2000 (see above) fixed 48 h after transfection with 1% formaldehyde for 15 min at room temperature and the reaction stopped by addition of 125 mM glycine for 5 min. The other steps of the ChIP experiments were performed according to the manufacturer’s instructions (Magna ChIP, Millipore). The extracts were immunoprecipitated with anti EGR1 (Cell signaling) or anti UBF antibodies (Santa Cruz). The samples obtained were analysed by PCR and qPCR. The products of PCR were analyzed on 2% agarose. Primers sequence used for PCR and real time analysis are: 47S rRNA promoter FW (5′-GTTTTTGGGGACAGGTGT-3′); 47S rRNA promoter Rev (5′-CCAGAGGACAGCGTGTCAGCA-3′); 18S Fw and 18S Rev, see above.

### Statistical Analysis

The analyses data have been described above. For all experiments analysis was carried out followed by post hoc comparision (ANOVA Scheffè F-test). Data were expressed as mean +/2 SEM.

## Results and Discussion

### EGR1 Colocalizes with Specific Nucleolar Markers in HeLa Cells

**Figure 1:**
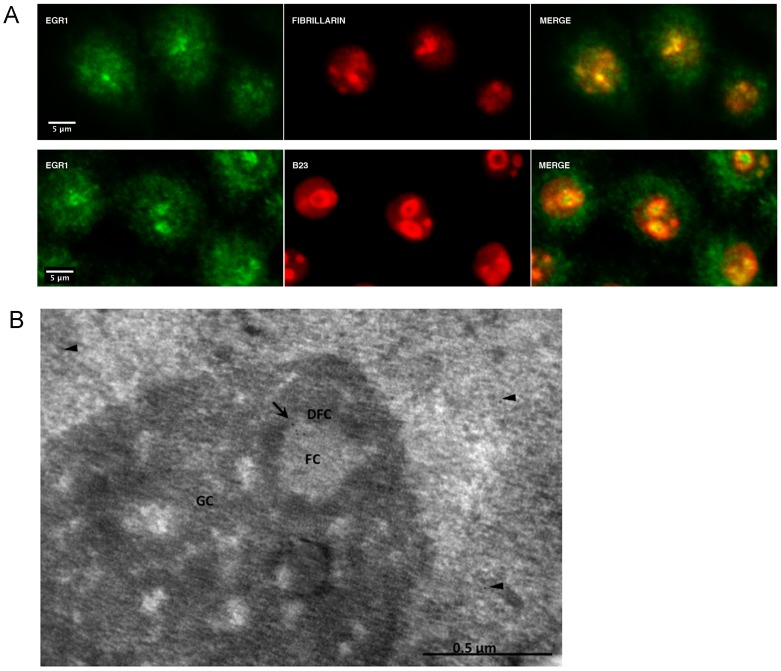
Nucleolar localization of endogenous EGR1. Imaging analysis. (A) Confocal images of EGR1 and fibrillarin (upper row) or EGR1 and B23 (lower row) in the nucleolus of HeLa cells. The images were obtained with a Leica SP2 and analyzed under HCX PL APO CS 63x. (B) Immunogold electron microscopy (EM) labeling of EGR1 in the fibrillar center of the nucleolus. Ultrathin sections of HeLa cells were embedded in Lowicryl K4M. (FC, Fibrillar center; DFC, Dense Fibrillar Component; GC, Granular Component. Arrow: labelling in the CF; arrowheads: labelling in the nucleoplasm).

When EGR1 is labeled with an antibody that recognizes the N-terminal region of the protein and is detected by immunofluorescence, an intense nuclear staining concentrated at relatively large nucleolar-like structures is observed in near 60% of the cells cultivated in 10% FBS or more than 90% of the cells cultivated in 0.2% FBS. Two specific nucleolar markers, fibrillarin and nucleophosmin/B23, colocalize with EGR1 in 90% and 30% of the cells, respectively, as assessed by confocal microscopy (Figure 1A). The specificity of EGR1 labeling within the nucleolus was tested by preincubating the antibody with an EGR1 peptide, effectively preventing the recognition of the endogenous protein in the nucleolar complex (Figure S1). Overlapping results were obtained with the human cell line 293T, carrying the expression of p53 WT, and the glioma cell line A172 (Figure S2 A and B), showing that the location of EGR1 to the nucleoli is not dependent on the specific cellular context. In this work for the first time EGR1 is recognized within the central region of the nucleolar compartment of cells grown either in 10% or 0.2% FBS (Figure 1A). This region corresponds to the fibrillar centers where the active sites of ribosomal RNA transcription are located [32], suggesting an involvement of the protein in ribosomal RNA transcription. For the sub-nucleolar localization we showed by immune electron microscopy on HeLa cells (Figure 1B) that EGR1 is found mostly in the fibrillar centers, particularly in the peripheral region near to the dense fibrillar component (Fig. 1B). Some immune gold labelling can be found in the nucleoplasm. It is interesting to note that this nucleolar labeling is consistent with the distribution of DNA, including rDNA, within the mammalian nucleolus.

However, we also found that EGR1 merges with B23 in a low fraction of cells. B23 is a nucleolar marker localized to the periphery of the nucleolus like ARF which EGR1 has been found to interact with (28). Whether these findings suggest for EGR1 an alternative site within the nucleolus, where it would exercises a biological role, it is matter of speculation.

**Figure 2:**
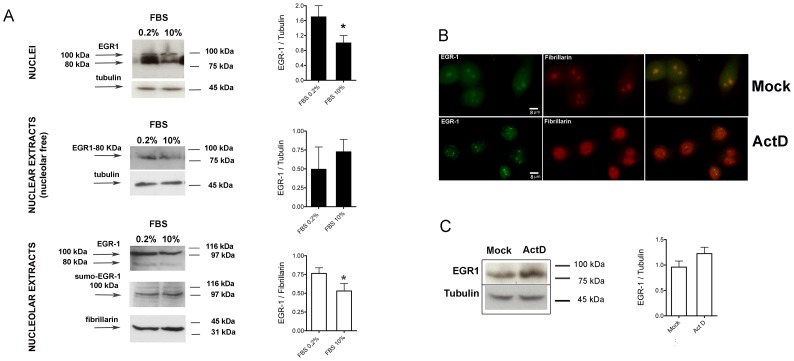
Nucleolar localization of endogenous EGR1. Biochemical evidences. (A) Detection by immunoblotting of EGR1 in nuclei, nuclear and nucleolar extracts of HeLa grown at 0.2% or 10% FBS. The sumoylated form of EGR1 present within the nucleolar extracts is detected by an anti-sumo1 antibody (Sigma-Aldrich). The band signals were quantitated for comparison between extracts of cells grown at 0.2% and 10% FBS. Signals were normalized to the loading control. Beta-tubulin and fibrillarin are shown as loading control. (B) Immunofluorescence of EGR1 after treatment with actinomycin D (0.04 µg/ml) for 1h at 37°C. HeLa cells were treated and stained for EGR1 and fibrillarin by immunofluorescence. The images were taken by under a 40X objective with a LEICA DM4000B. The pictures at the right show the merging of the two fluorescing proteins. (C) Nuclei of HeLa cells were extracted and immunoblotted to quantitate the expression of EGR1 following Actinomycin D treatment. Both immunofluorescence and western blotting show that the endogenous levels of EGR1 are not significantly affected by the treatment. Representative results of at least three separate experiments are shown. Comparison tests were assessed by one way ANOVA, and significances are shown where applicable. Asterisk (*) represent p≤0.05 when compared to relative controls.

We therefore performed western blotting of crude nuclei, nuclear (nucleolar-free) and nucleolar extracts of HeLa cells grown in normal and serum deprived conditions (Figure 2A). EGR1 from crude nuclei is resolved as two bands of 100 kDa and 80 kDa, as expected. The expression in cells grown at 0.2% FBS is twice the expression at 10% FBS. EGR1 in nucleolar-free nuclear extracts appears as a single band of 80 kDa, while the EGR1 from the nucleolar compartment is detected mostly as a 100 kDa molecule. This corresponde to the sumoylated form of the protein [28], as confirmed by staining with an anti-sumo1 antibody. A quantitative comparison of the nucleolar 100 kDa isoform at the two different serum concentrations shows that EGR1 is significatly more abundant in cells grown at 0.2% FBS. The measure of the expression levels of EGR1 in all three extracts has been taken in at least three independent experiments.

HeLa cells treated with a low dose of actinomycin D (0.04 µg/ml actinomycin D), which inihibits mainly the RNA polymerase I dependent rRNA transcription [33] have less EGR1 in the nucleolus. In fact EGR1 is detected by immunofluorescence at its localization likely because after treatment it is relocated with fibrillarin into the nuclear cups shown in Figure 2B. This suggests that EGR1 localization to the nucleolus is dependent, at least in partially, on ongoing transcription of rRNA in intact nucleoli.

### The Nucleolar Localization of EGR1 is Specified by the C-terminal Region

**Figure 3:**
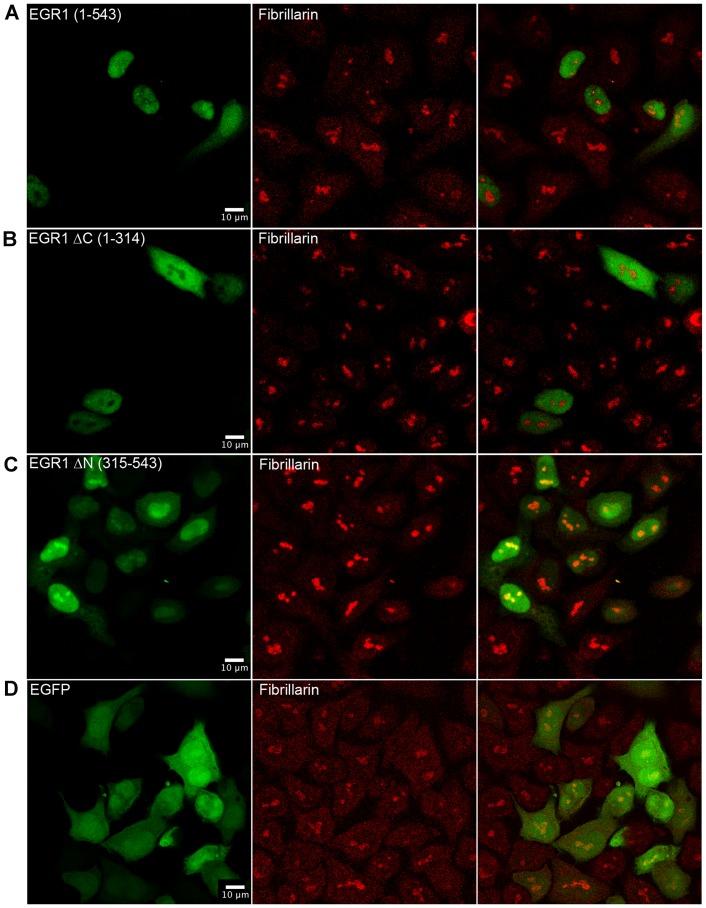
The C-terminal region of EGR1 contains the nucleolar localization sequence. Confocal images of HeLa cells transfected with (A) the full length EGR1 (1–543 AA), (B) the N-terminal (ΔC-EGR1) (1–314 AA), (C) the C-terminal EGR1 (ΔN-EGR1) (315–543 AA). Each construct was fused to the GFP. (D) Empty pEGFP vector. Full length EGR1, N-terminal EGR1, C-terminal EGR1 and stained with an antibody to fibrillarin.

HeLa cells were transfected with plasmids expressing either the full length or two truncated forms of EGR1 fused with the N-terminal region of GFP. Protein expression was monitored by confocal microscopy. The first deletion construct contained the N-terminal region of EGR1 from amino acid 1 to 314 including the transactivation (1–281 AA) and the repression domains (282–315 AA). The second fusion protein contained the C-terminal region of EGR1 from amino acid 315 to 543 including the DNA binding domain (338–418 AA). The C-terminus but not the N-terminus (Figure 3C and 3B, respectively) nor the GFP alone (Figure 3D) colocalized with fibrillarin and showed strong nucleolar staining. The full EGR1/GFP fusion protein did localize mostly in the nucleus and it is associated with the nucleolus (Figure 3A). Thus, the N-terminus deprived EGR1-GFP fusion protein has apparently a greater ability to accumulate to the nucleolus than the full lenght protein. This might be a consequence of the less complex structure of the truncated EGR1 polypeptide. It is conceivable that the relocation of EGR1 to the nucleolus is subjected to regulation by an array of cognate molecules physically interacting with yet unknown domains spread over the EGR1 molecule. If this is true, a smaller EGR1 would have a lower binding valence and, in turn, less obligations limiting the EGR1 localization to the nucleolus. These results suggest that the C-terminal region is important for promoting the association of EGR1 with nucleolar components, and that a specific nucleolar localization signal may reside in this region of the protein. To address this hypothesis EGR1 protein was analysed with a specific program (NoD, Nucleolar localization sequence detector available at http://www.compbio.dundee.ac.uk) and a NoLS sequence between residues 408 and 430 was found, within the third zinc finger of the protein. It has been suggested recently that nuclear factors containing a zinc finger DNA binding domain may actually localize to the nucleolus and have the ability to bind to RNA [34]. Indeed, p53 localizes to the nucleolus and binds to RNA with the C-terminal portion [35]. Interestingly, the C-terminal region of EGR1 is important for the interaction with ARF that in turn is involved in sumoylation of EGR1 itself [28].

### EGR1 Interferes with the Production of 47S rRNA Precursor

**Figure 4:**
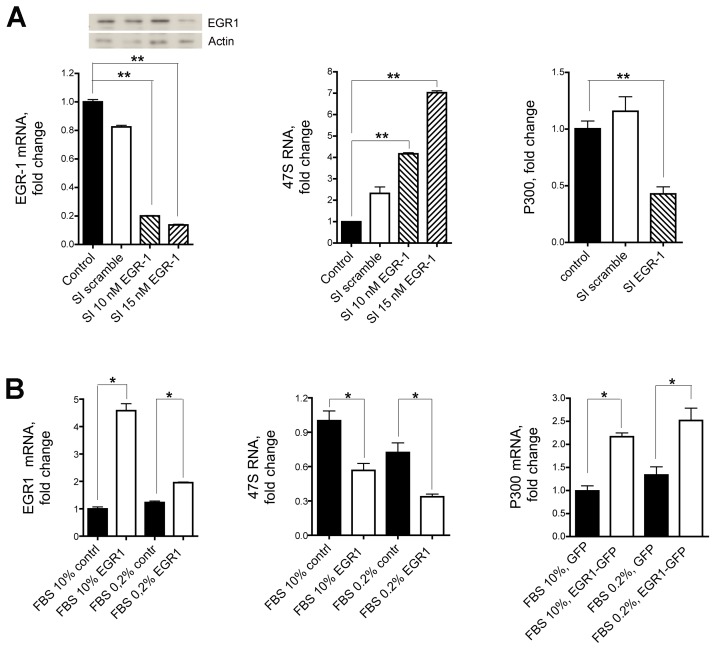
47S rRNA and EGR1. (A) The synthesis of 47S rRNA is strongly upregulated following inhibition of EGR1. 47S synthesis in Hela cells grown in 0.2% FBS is significantly increased following endogenous EGR1 silencing with either 10 nM or 15 nM specific siRNA (middle graph). 47S synthesis is not affected in cells treated with a scrambled sequence compared to the untreated cells taken as control. The levels of expression of EGR1 and p300 following the siRNA treatment are shown in the left and right graphs, respectively. Both levels are significantly diminished after EGR1 silencing. (B) 47S synthesis in HeLa cells grown in 0.2% or 10% FBS is significantly depressed after transfection of full length EGR1. The levels of expression of EGR1 and p300 are shown in the left and right graphs, respectively. As expected, both levels are significantly upregulated after EGR1 transfection compared to control cells. Representative results of at least three separate experiments are shown. Comparison tests were performed by one way ANOVA, and significant results are highlighted with asterisks (* p≤0.05, ** p≤0.01 in comparison with relative controls).

RNA polymerase I directs the transcription of the 47S rRNA genes, a class of genes found in multiple copies in the nucleoli of eukaryotic cells. To test whether EGR1 regulates the biogenesis of ribosomes, HeLa cells were either incubated with EGR1 specific siRNA (Figure 4A) or transfected with a vector carrying a full length EGR1 cDNA (Figure 4B). The use of siRNA specific for EGR1 but not of a scrambled control oligonucleotide effectively suppresses the level of EGR1 (Figure 4A) and slightly reduces its staining within the nucleolus (Figure S3).

**Figure 5:**
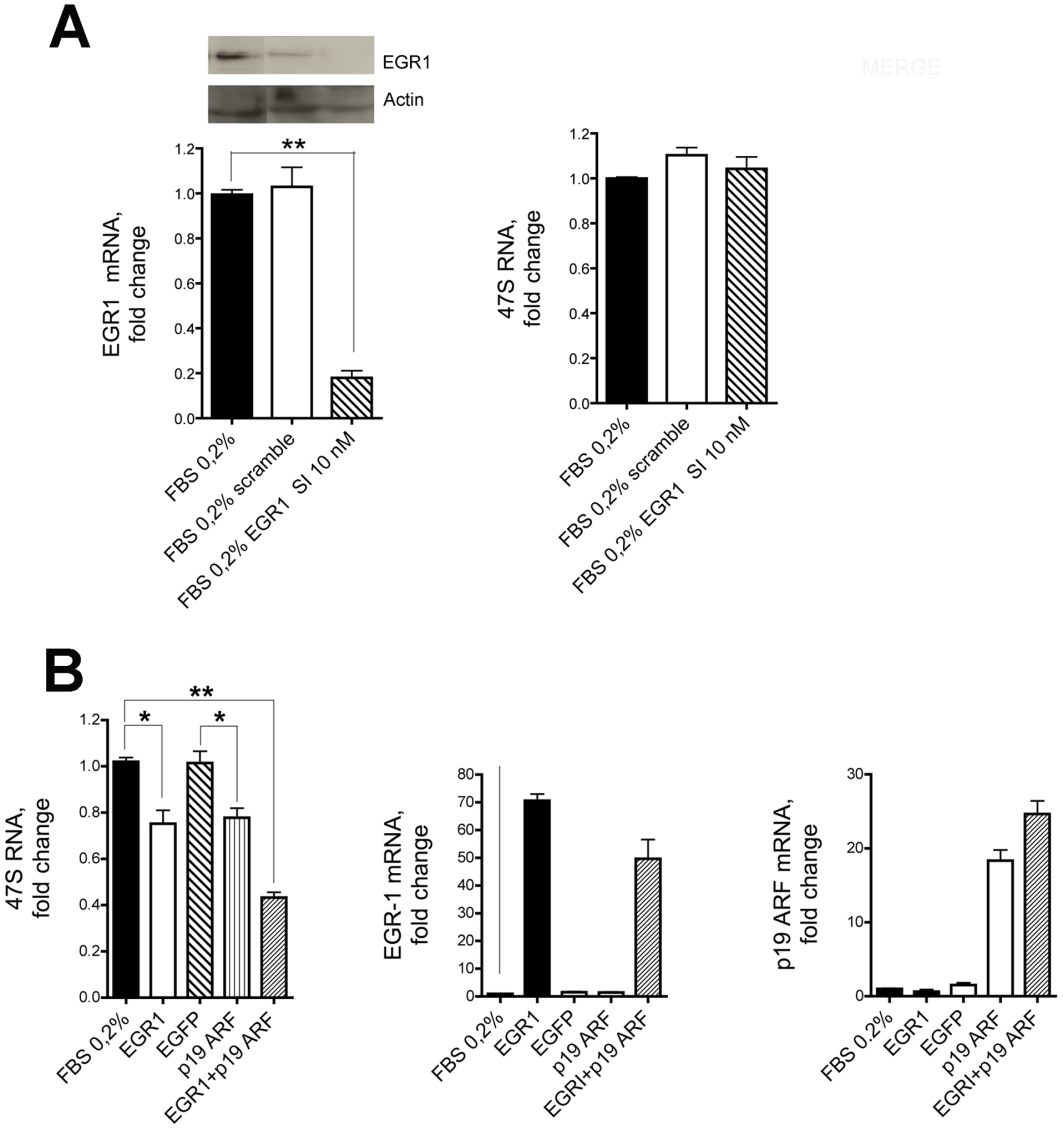
EGR1 downregulates the expression of 47S rRNA in association with ARF. (A) The synthesis of 47S rRNA in NIH 3T3 (ARF−/−) cells grown in 0.2% FBS (right graph) is not affected when the expression of endogenous EGR1 is silenced with 10 nM of specific siRNA (left graph). (B) Viceversa, the synthesis of 47S rRNA (left graph) is greatly reduced when both EGR1 (middle graph) and p19ARF genes (right graph) are transfected and expressed in NIH 3T3 following transfection with plasmid expression vectors. Transfection with pEGFP is shown as control. Comparison tests were assessed by one way ANOVA, and significant differences are highlighted with asterisks (* p<0.05; ** p<0.01).

We also observed that the levels of 47S rRNA precursor correlated inversely with the expression of EGR1 and that of p300, a known target of EGR1 [36]. These results could be successfully replicated with the 293T cell line (Figure S2 C). On the other hand, the endogenous EGR1 is not localized to the nucleolus in cells deficient for ARF such as NIH 3T3 [37] and silencing with siRNA EGR1 does not alter the 47S pre-rRNA levels (Figure 5A). Transfection of these cells with full length EGR1 has only a limited effect on the expression of the 47S pre-rRNA levels. However, when NIH 3T3 are cotransfected with plasmids containing both the full length EGR1 and ARF cDNA, the levels of 47S ribosomal RNA are significantly downregulated up to 60% (Figure 5B). Consistently with our results, *ARF −/−* mice do not exhibit sumoylated forms of EGR1 [28], display significant alterations of nucleolar morphology and abundance, and have higher level of rRNA transcription compared to *wild-type* animals [38].

We do not know yet the mechanism by which variation in EGR1 expression is linked to suppression of ribosomal precursor synthesis. For example one can speculate that EGR1 may interfere with rRNA transcription. Very preliminary results show that following EGR1 silencing in HeLa cells only the 47S pre-RNA is increased but no other ribosomal precursors, suggesting that the control of EGR1 over ribosomal RNA synthesis might be exercised through an enhacement of RNA polymerase I activity (data not shown).

### EGR1 Affects the RNA Polymerase I Activity by Interacting with UBF

**Figure 6:**
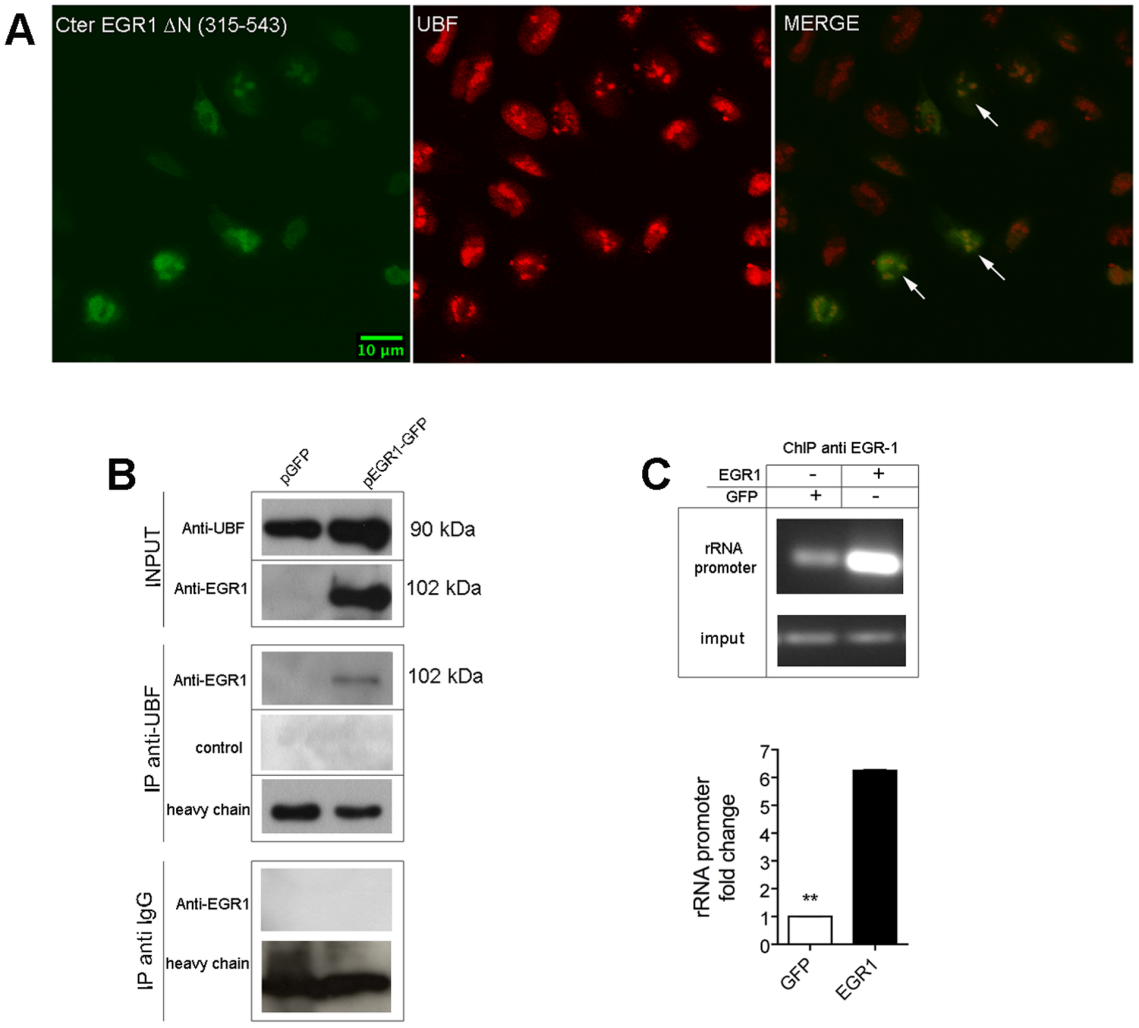
EGR1 binds to UBF in HeLa cells. (A) Confocal analysis of Hela cells transfected with the C-terminal EGR1 (ΔN 1–314) shows the colocalization of EGR1 fragment with UBF. (B) Extracts (150 µg) of HeLa cells transfected with full length EGR1-GFP are immunoprecipitated with an antibody to UBF. (C) Chromatin precipitation assay. DNA fragments of ribosomal RNA promoter are immunoprecipitated with an antibody to EGR1 from extracts of HeLa cells transfected with full length EGR1. A six fold enrichment in ribosomal RNA promoter fragments was obtained from extracts of transfected cells compared to mock extracts. Representative results of at least three separate experiments are shown. Comparison tests were performed as above described (** p≤0.01).

The above experiments strongly suggest that EGR1 participates in the pre-rRNA synthesis. UBF, together with SL1, is a key regulator of RNA polymerase I and pre-rRNA synthesis [39] by binding to upstream control elements and core sequences within the rRNA promoter, and by directly associating with the RNA polymerase I. It is possible that EGR1 could exert an inhibitory role on the RNA polymerase by interacting with UBF, a known partner of the RNA polymerase I. To test this idea, we transfected HeLa cells with the C-terminus-EGR1/GFP construct (ΔN 315–543 AA), and found by confocal analysis that EGR1 colocalizes with UBF in the nucleolus (Figure 6A). This fusion protein colocalizes better than the full length EGR1/GFP protein. Furthermore, by applying an anti-UBF antibody to lysates of HeLa cells transfected with the full length EGR1 expression vector, we observed that EGR1 co-immunoprecipitates with UBF (Figure 6B). It is possible that EGR1 interferes with the activity of RNA polymerase I by interacting with the ribosomal promoter, either directly or indirectly. To test this idea we have first performed a chromatin precipitation assay where DNA fragments of ribosomal RNA promoter were incubated in presence of extracts of HeLa cells either transfected with full length EGR1 or mock extracts. As clearly shown in Figure 6C, following EGR1 overexpression we observed a six fold enrichment in ribosomal RNA promoter fragments, compared to the control. It is already known that UBF is stably associated with the ribosomal promoter [40]. The above results may suggest that the interaction between EGR1 and UBF would take place on ribosomal promoter itself leading to a disregulation of ribosomal RNA transcription. Currently we are working to test this hypothesis. An interaction with UBF may have destabilizing effects of the UBF-SL1 complex. Similar mechanisms have been found with other UBF-interacting tumor suppressors, such as pRB or p130. They have been shown to interact with UBF directly and to inhibit the recruitment of cofactors required for rRNA transcription [41]. Also p53 interferes with the RNA polymerase I activity by interacting with SL1 [42].

## Conclusions

We suggest that nucleolar localization of EGR1 occurs via its C-terminal region and this event requires the interaction with ARF. In the nucleolus EGR1 binds to UBF and contributes to the regulation of RNA polymerase I activity in fact the levels of the 47S rRNA precursor fluctuate up or down following EGR1 silencing or overexpression, respectively. We hypothesize that EGR1 by binding to 47S rRNA prevents UBF from interacting with the rDNA promoter and thus the initiation complex formation.

All together our findings point to a new mechanism for EGR1 as a regulator of cell proliferation.

## Supporting Information

Figure S1 **Specificity of immunofluorescence staining of endogenous EGR1 in HeLa cells.** (A) The anti-EGR1 specific antibody is first reacted with a blocking peptide (Cell Signaling cod. 1015) carrying an immunogenic sequence from the N-terminal portion of EGR1 protein, and then incubated with the fixed cells. (B) Control staining with unadsorbed anti-EGR1 specific antibody.
(PDF)

Figure S2 **Endogenous EGR1 localizes to the nucleolus of 293T and A172 cell lines, affecting the level of 47S precursor rRNA.** EGR1 colocalizes with fibrillarin in the nucleolus of 293T (A) and the glioma cell line A172 (B). EGR1, 47S and p300 RNA expression in 293T cells treated with 15 nM siRNA EGR1 (C).
(PDF)

Figure S3 **Immunofluorescence of endogenous EGR1 in HeLa cells after EGR1 silencing.** (A) Cells treated with scramble control oligonucleotides (A) or (B) 15 nM siRNA specific for EGR1.
(PDF)
