# Supplementary figures and images for: The Transcription Factor EGR1 Localizes to the Nucleolus and Is Linked to Suppression of Ribosomal Precursor Synthesis
Source: PLoS One. 2014 May 1;9(5):e96037. doi: 10.1371/journal.pone.0096037 (PMC4006901; doi:10.1371/journal.pone.0096037)

A

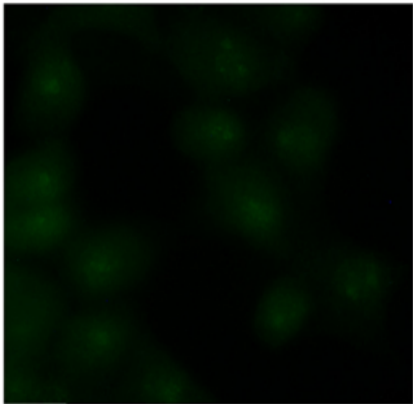

B

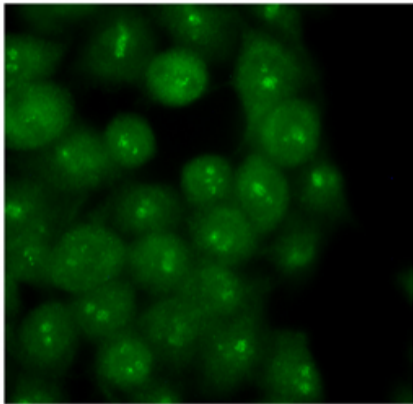

FIG.S1

Supplement: Figure S1 — Specificity of immunofluorescence staining of endogenous EGR1 in HeLa cells. (A) The anti-EGR1 specific antibody is first reacted with a blocking peptide (Cell Signaling cod. 1015) carrying an immunogenic sequence from the N-terminal portion of EGR1 protein, and then incubated with the fixed cells. (B) Control staining with unadsorbed anti-EGR1 specific antibody. (PDF) [file pone.0096037.s001.pdf]

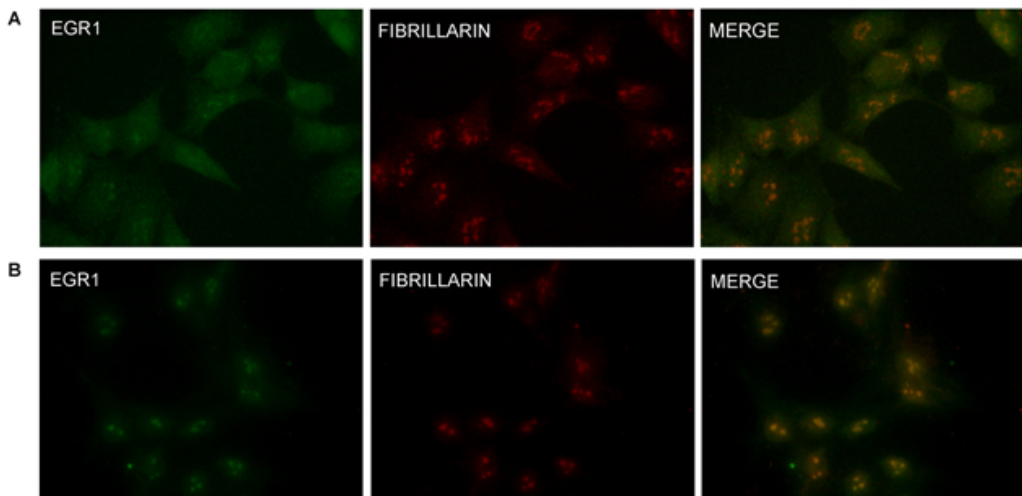

**C**

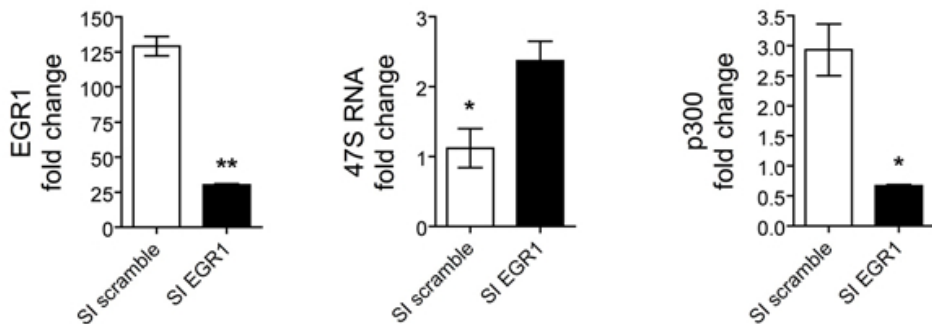

**FIG.S2**

Supplement: Figure S2 — Endogenous EGR1 localizes to the nucleolus of 293T and A172 cell lines, affecting the level of 47S precursor rRNA. EGR1 colocalizes with fibrillarin in the nucleolus of 293T (A) and the glioma cell line A172 (B). EGR1, 47S and p300 RNA expression in 293T cells treated with 15 nM siRNA EGR1 (C). (PDF) [file pone.0096037.s002.pdf]

A

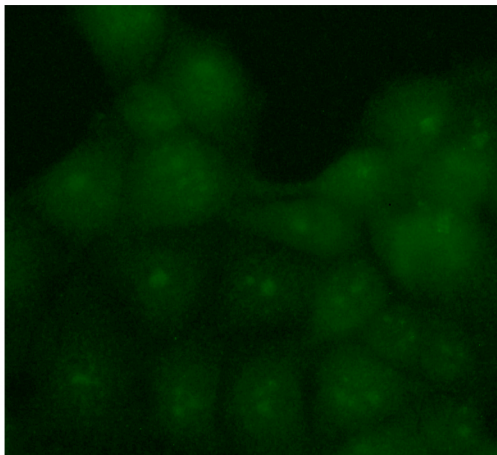

B

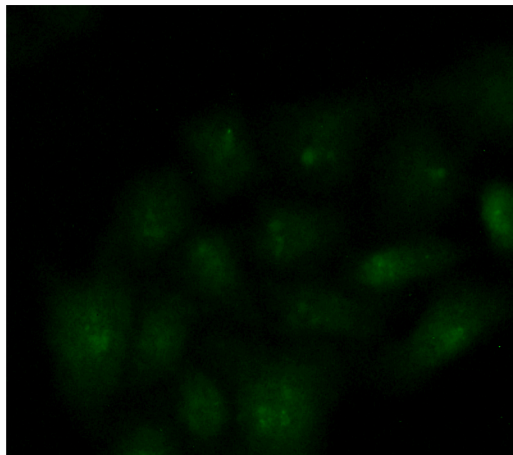

Fig. S3

Supplement: Figure S3 — Immunofluorescence of endogenous EGR1 in HeLa cells after EGR1 silencing. (A) Cells treated with scramble control oligonucleotides (A) or (B) 15 nM siRNA specific for EGR1. (PDF) [file pone.0096037.s003.pdf]
